# A Treatise on the Diseases of the Eye

**Published:** 1883-09

**Authors:** 


**A Treatise on the Diseases of the Eye.** By J. LOELBERG WELLS, F. R. C. S. Edited by CHARLES STEDMAN BULL, A. M., M. D.

The early date at which another edition of this work has appeared is a good indication of the esteem in which it is very properly held. With so many text-books on the same subject it is hard to make invidious comparisons; but with due regard to the thorough work of German authors, it is not too much to say that Wells' work is the best book on the eye, for students and practitioners, that is now available to English readers. The new editions, issued from time to time, during the last fifteen years, have been under such able management as to keep up the standard of worth at first attained. In its present form, we have a practical exposition of the latest views concerning this class of diseases; and much credit is due to Dr. Bull for the careful study of current literature, in order to present an abstract of all that is new and worthy of mention.

